# Multi-analyte liquid biopsy approaches for early detection of esophageal cancer: the expanding role of ctDNA

**DOI:** 10.3389/fonc.2025.1622984

**Published:** 2025-08-14

**Authors:** Derek Tai, Kareem Latif, Pranati Shah, Daniel Park, Christiana Crook, Sofia Guzman, Gagandeep Brar, Daneng Li, Dani Castillo

**Affiliations:** ^1^ Department of Internal Medicine, Loma Linda University Medical Center, Loma Linda, CA, United States; ^2^ Department of Internal Medicine, University of California San Francisco Fresno, Fresno, CA, United States; ^3^ Department of Medical Oncology and Therapeutics Research, City of Hope, Duarte, CA, United States

**Keywords:** CtDNA, esophageal cancer, early detection, liquid biopsy, biomarker, esophageal adenocarcinoma, esophageal squamous cell carcinoma, surveillance

## Abstract

Circulating tumor DNA (ctDNA) has emerged as a promising biomarker for the early detection of esophageal cancer (EC), offering a minimally invasive means to assess tumor-derived genomic and epigenomic alterations. This review synthesizes current data on ctDNA biology, detection technologies, diagnostic performance, and clinical applicability in both esophageal adenocarcinoma and squamous cell carcinoma. We conducted a comprehensive literature review of PubMed-indexed studies on ctDNA in EC, emphasizing recent (January 1, 2019– December 31, 2024) findings, systematic reviews, and meta-analyses. Key data on ctDNA characteristics, diagnostic performance in both early-stage esophageal adenocarcinoma and squamous cell carcinoma, and clinical outcomes were extracted. We also discuss technical and clinical challenges in ctDNA assays and future perspectives for integrating ctDNA evaluation into clinical practice. We highlight technological innovations such as methylation profiling, fragmentomics, and ultrasensitive sequencing, and compare ctDNA-based approaches to alternative non-endoscopic modalities. While early studies report encouraging sensitivity and specificity for ctDNA in high-risk populations, specifically with methylation assays, current data remain limited by small sample sizes, retrospective design, low tumor DNA abundance, and heterogeneity in assay methodology. Furthermore, the clinical implementation of ctDNA-based screening must address population-level feasibility, cost-effectiveness, and health equity. We conclude that ctDNA holds significant potential for early EC detection but remains investigational pending validation in large prospective cohorts.

## Introduction

Esophageal cancer (EC) is a highly aggressive malignancy with poor overall survival, largely because most cases are diagnosed at advanced stages. Globally, EC ranks among the top ten cancers in both incidence and mortality​ (1). Despite therapeutic advances, the prognosis remains grim: overall 5-year survival is only about 20%, and falls below 10% in patients diagnosed with metastatic disease​. Early-stage EC is often asymptomatic or causes only mild, nonspecific symptoms, leading to diagnostic delays​ (2). As a result, the majority of patients present with locoregionally advanced or metastatic tumors, which contributes to the high mortality rate​ (3, 4). Early detection of EC is critical, as it offers the opportunity for curative treatment (endoscopic therapy or surgery) that may not otherwise be available in advanced stages. For example, patients with tumors confined to the mucosa or submucosa can achieve survival rates far exceeding those of patients with advanced disease (5, 6). However, current early detection strategies are limited. Upper endoscopy with biopsy is the gold standard for diagnosing EC and its precursor lesions, but endoscopic screening of at-risk populations, such as those with Barrett’s esophagus or heavy tobacco/alcohol use in regions prone to squamous cell carcinoma, is invasive, costly, and difficult to implement for widespread screening (7, 8). Unlike some other gastrointestinal malignancies, no blood-based biomarker is available for the early diagnosis or screening of EC (9, 10). Serum protein markers such as CEA or CA19–9 lack sensitivity and specificity for esophageal malignancies. This creates an urgent need for novel, non-invasive biomarkers that could aid in the detection of early esophageal neoplasia.

Circulating tumor DNA (ctDNA) has emerged as a compelling candidate in EC screening and detection. ctDNA refers to fragments of tumor-derived DNA that circulate in the bloodstream as a subset of the cell-free DNA (cfDNA) pool (7). These fragments carry tumor-specific genetic and epigenetic alterations, such as somatic mutations, copy number changes, and DNA methylation patterns, mirroring the molecular profile of the cancer. Because ctDNA can be sampled through a blood draw, it offers a non-invasive, real-time window into the cancer status of a patient​ (11–13). In several malignancies, ctDNA assays are already used clinically for mutation testing, disease monitoring, and recurrence detection (14–19). In EC, interest in ctDNA has grown as studies have begun to demonstrate its potential utility in diagnosing tumors earlier, monitoring treatment response, and detecting minimal residual disease (20). Notably, ctDNA could fill the lack of a blood-based biomarker for EC, complementing or potentially triaging the use of endoscopy in screening and surveillance (21).

## Objectives/methods

**Figure 1:**
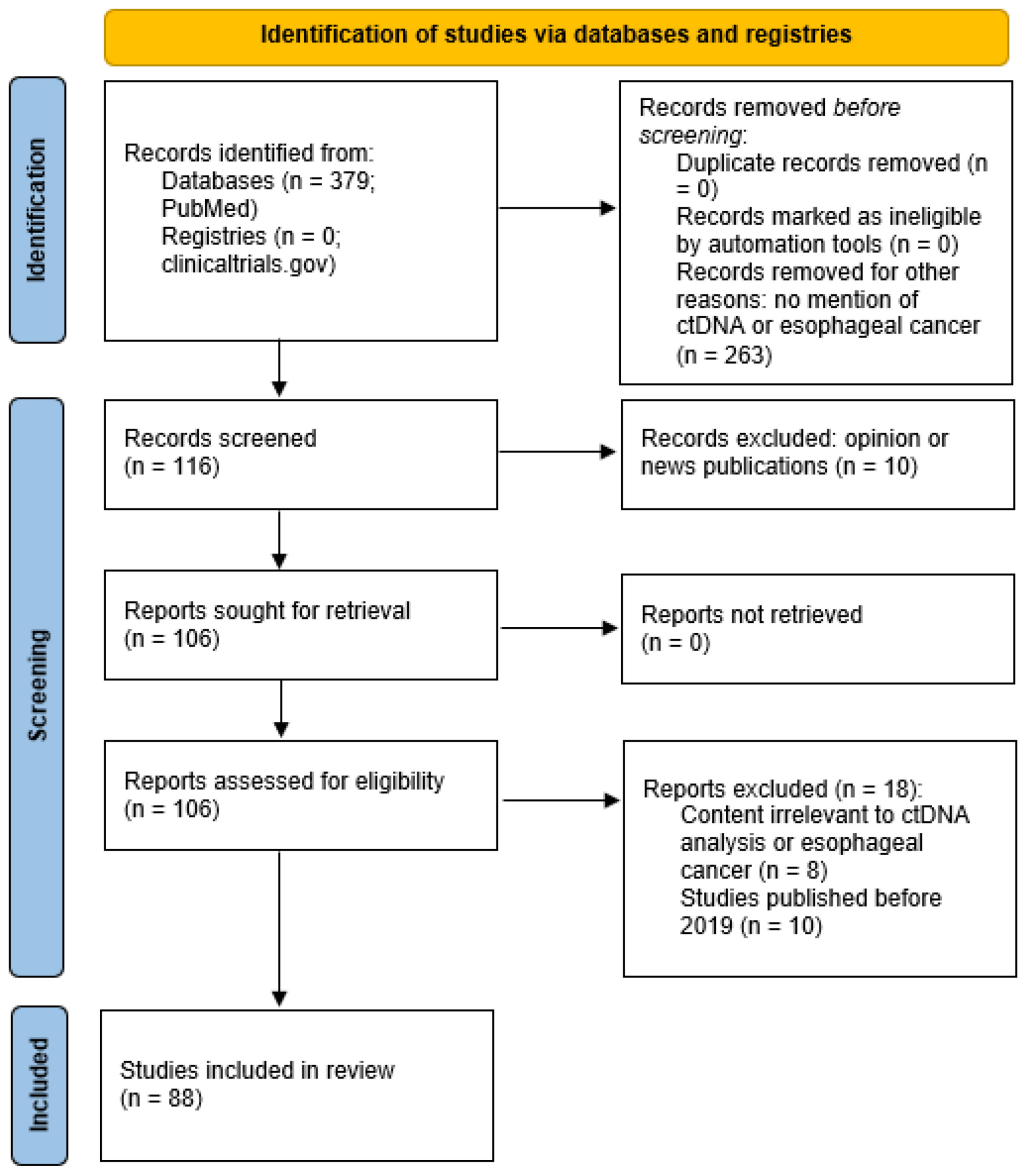
PRISMA 2020 flow diagram of published works relating to ctDNA and esophageal cancer.

This review provides a comprehensive overview of the use of ctDNA in serum plasma for early detection of EC. We first outline the biological characteristics of ctDNA relevant to cancer detection. We then examine evidence for the clinical utility of ctDNA in identifying early-stage EC and summarize findings from key systematic reviews and meta-analyses. We discuss the technical and clinical challenges of integrating ctDNA into standard of care treatment of EC. Finally, we explore future perspectives on improving ctDNA assays and integrating them into clinical practice and standard of care, including screening high-risk populations and implementing early intervention strategies. The focus of this review is on early-stage detection and clinical utility while also considering relevant technical advances. Evidence from peer-reviewed studies is highlighted to inform the current state and future direction of this rapidly evolving field. **Figure 1** shows the PRISMA Flow Diagram utilized for database and registry search of relevant records pertinent to this review.

## Biology and characteristics of ctDNA

### Definition and origins

**Figure 2:**
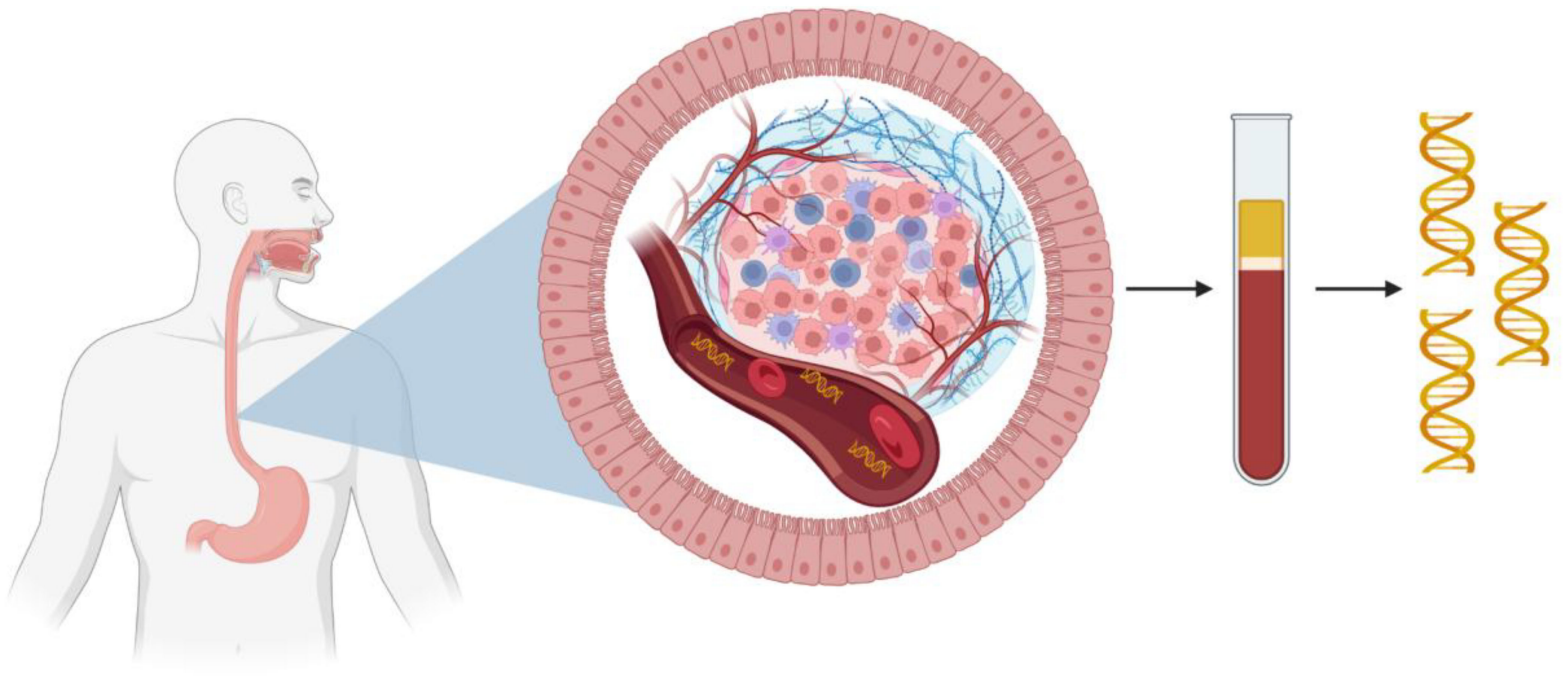
Release of ctDNA in esophageal adenocarcinoma.

ctDNA is defined as the fraction of cfDNA in the bloodstream that originates from cancer cells. Normal cfDNA is released primarily from apoptosis and turnover of non-malignant cells, a recognized phenomenon since the 1940s​ (22–25). In cancer patients, a proportion of cfDNA fragments derive from tumor cell apoptosis, necrosis, and active secretion, carrying tumor specific alterations such as somatic mutations and methylation changes as demonstrated by Stroun et al​ (7, 25). Importantly, ctDNA is present in the bloodstream of patients with EC and other solid tumors, though typically as a small fraction of total cfDNA, especially in early-stage disease​ (26). **Figure 2** shows an example of how ctDNA may be released from esophageal adenocarcinoma (EAC) into circulation. ctDNA concentrations and tumor fraction correlate with disease burden where levels tend to be elevated in advanced EC due to higher tumor turnover and levels tend to be lower, sometimes below the detection threshold of standard assays, in localized disease​ (27, 28).

### Physical characteristics

ctDNA fragments are typically short with modal lengths around 134–145 base pairs (bp), compared to roughly 165 bp from healthy cells (29). This difference in fragment size distribution is attributed to the differing nuclease digestion patterns in apoptotic tumor cells. The shift in fragment size distribution can be utilized in ctDNA detection such as in fragmentomics. ctDNA is rapidly cleared from circulation with a half-life ranging from minutes to a few hours due to degradation by DNases and uptake by the liver, spleen, and kidneys (30–33). This short half-life allows ctDNA to reflect real-time tumor dynamics but also requires strict pre-analytical handling. Blood must be collected in specialized tubes and processed promptly to avoid contamination from lysed white blood cells.

### Genomic and epigenomic content

**Figure 3:**
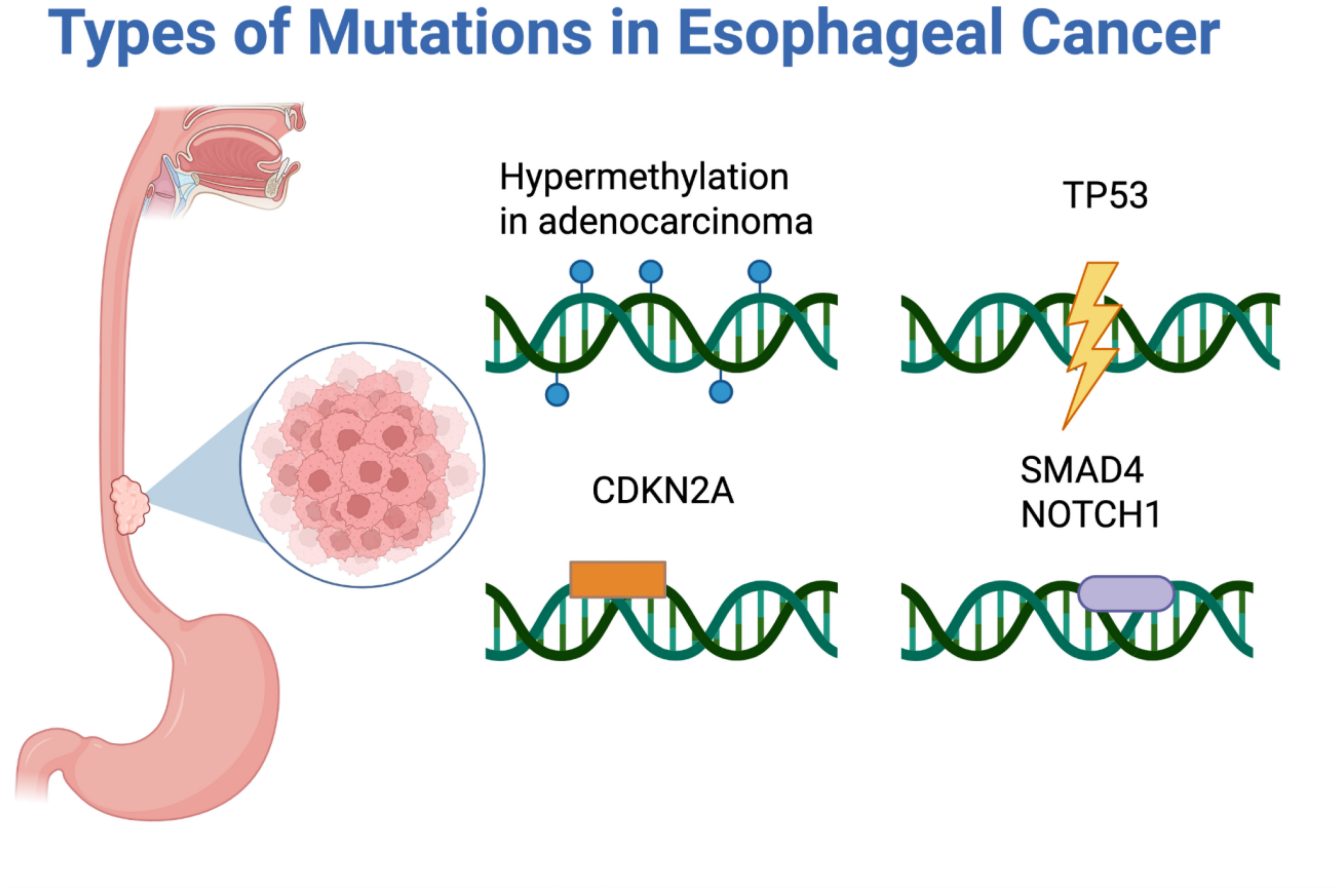
Types of mutations seen in esophageal cancer: hypermethylation in adenocarcinoma, *TP53*, *CDKN2A*, *SMAD4*, and *NOTCH1*.

ctDNA carries tumor-specific mutations and epigenetic changes reflective of its tissue origin (7). In EC, common genomic alterations include mutations in *TP53*, found in both EAC and esophageal squamous cell carcinoma (ESCC), as well as *CDKN2A*, *SMAD4*, and *NOTCH1* as seen in **Figure 3** (34). In addition to genomic mutations, esophageal tumors, particularly EAC, show widespread DNA hypermethylation early in carcinogenesis (35). These epigenetic alterations represent abundant, chemically stable markers in ctDNA, making them ideal biomarkers for early detection​ (36, 37). For example, methylation of tumor suppressor genes in EC such as *SEPTIN9*, *TFPI2*, *FHIT*, and *RASSF1A* have been studied as ctDNA markers​ and utilized in multi-gene methylation panels, enhancing sensitivity compared to single mutation assays (38, 39).

### Detection technologies

**Figure 4:**
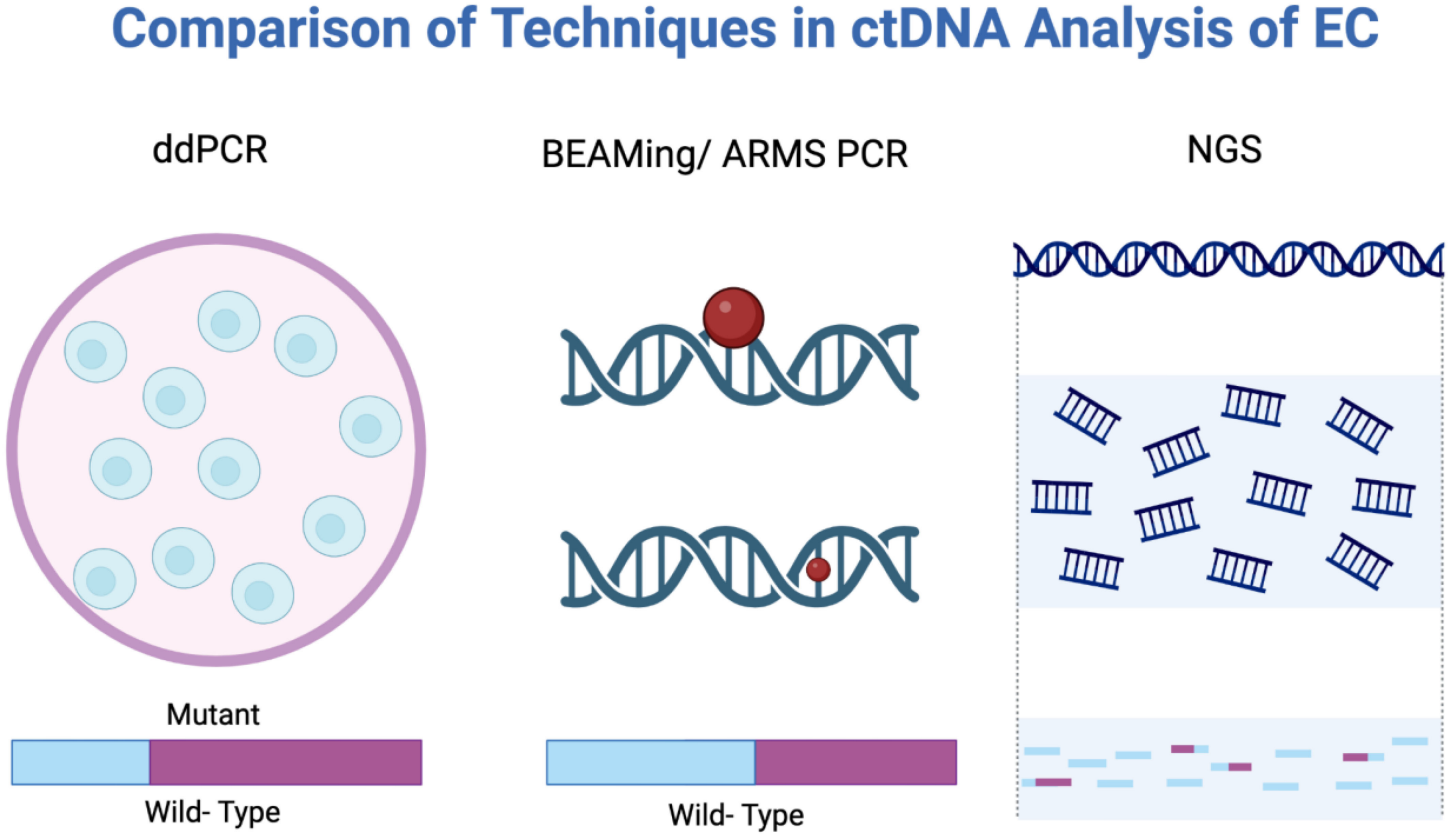
Analytical methods used in ctDNA analysis: ddPCR, BEAMing/ARMS PCR, and NGS. BEAMing/ARMS PCR, Beads, Emulsion, Amplification, Magnetics/Amplification Refractory Mutation System polymerase chain reaction; ddPCR, Digital droplet polymerase chain reaction; NGS, Next-generation sequencing.

Two major technological approaches are used to detect ctDNA: polymerase chain reaction (PCR) and next-generation sequencing (NGS)​ (40, 41). **Figure 4** provides a visual comparison of digital droplet PCR (ddPCR), Beads, Emulsion, Amplification, Magnetics/Amplification Refractory Mutation System (BEAMing/ARMS) PCR, and NGS. ddPCR or BEAMing/ARMS PCR techniques allow quantification of known mutations or methylation sites with single-base specificity, detecting mutant allele fractions as low as 0.01%​ (42). However, this requires prior knowledge of the tumor’s mutation profile, typically garnered from a tumor biopsy, limiting utility in asymptomatic or screening contexts.

NGS-based methods, including targeted panels, whole-exome/genome sequencing, and error-corrected sequencing techniques, can identify a broad array of mutations or methylation changes without prior knowledge of the tumor’s genotype​ (43, 44). While NGS can detect ctDNA at mutant allele fractions around 0.1% or lower, they are generally less sensitive than ddPCR but provide richer molecular data including copy number variation, fragmentation patterns, and methylome-wide changes, improving sensitivity for detecting cancer-derived DNA​ (45). However, broader sequencing with NGS may increase the risk of detecting incidental findings from clonal hematopoiesis of indeterminate potential (CHIP), confounding interpretation (46, 47).

Regardless of the method, a major limitation in early EC detection is the low ctDNA levels, especially in early-stage ECs with tumors less than 2 cm or T1 staging. One systematic review noted minimal sensitivity for lesions less than 1 cm (48). Thus, while ctDNA is an attractive biomarker, its effective use in early detection requires ultrasensitive techniques and potential multi-modality approaches such as combining genomic mutations, methylation markers, and even protein markers to capture weak ctDNA signals in early-stage EC.

## Clinical utility of ctDNA in early esophageal cancer detection

### Detection of primary tumors

Multiple studies have demonstrated the feasibility of ctDNA as a diagnostic biomarker in EC. Egyud et al. first reported successful ctDNA detection in plasma from patients with EAC, laying down a foundation as a proof of concept for further studies (49). More recently, Min et al. summarized the role of ctDNA in distinguishing EC from benign lesions with reasonable sensitivity and specificity (50). A prospective study by Qin et al. evaluated a targeted five-gene methylation panel, which detected ctDNA in EC with about 74% sensitivity and 91% specificity, including roughly 43% of stage I and 64% of stage II cases​ (51). A 2022 meta-analysis of ctDNA-based EC diagnostic studies reported a pooled sensitivity of 71.0% (95% confidence interval [CI] 55.7%–82.6%) and specificity of 98.6% (95% CI 33.9%–99.9%), emphasizing its low false positive rate and potential role as a screening tool in high-risk populations​ (21). However, the moderate sensitivity in early-stage disease limits its standalone utility. In clinical practice, ctDNA could be used to prioritize high-risk individuals with Barrett’s esophagus, tylosis, or heavy tobacco/alcohol use, expediting an endoscopic evaluation.

### Performance in early-stage disease

ctDNA detection correlates strongly with tumor stage. Advanced ECs, stages III–IV, reliably shed detectable ctDNA, whereas early-stage tumors, stage I or carcinoma *in situ*, often fall below detection thresholds. In a case-control study using methylation sequencing, overall sensitivity was 75% for advanced EC and 58.8% for stage 0–II EC​ (52). Similarly, other methylation classifiers showed sensitivity as low as 0%–20% in patients with stage I EC, rising to 60%–75% in stage II EC (53).

A study by Liu et al. combined three-gene methylation markers, *SEPTIN9, TFPI2*, and *FHIT*, and reported an overall sensitivity of 75%–79% sensitivity for stage I–II ECs, representing an improvement over earlier studies and approaching the performance of other minimally invasive tools such as Cytosponge (53). Additionally, emerging multi-omic assays are showing that integrating cfDNA 5-hydroxymethylcytosine (5hmC) with copy number analysis achieved a greater than 80% sensitivity and 88% specificity in detecting ESCC, with potential to identify high-grade dysplasias​ (45).

### Comparisons to other modalities

ctDNA-based detection should be considered alongside other emerging non-endoscopic approaches. One alternative in development is targeted esophageal sampling, specifically the Cytosponge, a minimally invasive device that samples esophageal cells for molecular analysis in patients with Barrett’s esophagus. While effective in detecting dysplasia via methylation markers, Cytosponge is limited to adenocarcinoma and still requires instrumentation.​ (54–56).

Breath testing for volatile organic compounds has shown modest ability to detect EC but remains investigational at the time of this review​ (57–59).

Other image-based modalities such as AI-assisted endoscopy, confocal laser endomicroscopy, and endocytoscopy offer high-resolution mucosal visualization. Other biomarkers such as microRNAs and salivary biomarkers are under investigation (60).

In contrast, ctDNA offers a fully blood-based test with potential to detect both EAC and ESCC, making it more universally applicable. Several multi-cancer blood tests, such as CancerSEEK, have begun including EC in its cancer detection assay. CancerSEEK combined 16 gene mutations and 8 protein markers to achieve 69%-77% sensitivity for EC overall, but only 20% in stage I EC, highlighting the continued challenge of early detection. Notably, specificity exceeded 99%, and localizing the organ of origin by machine learning suggests that positive results could direct follow-up endoscopy for further evaluation​ (8). Future EC-specific ctDNA tests may be combined with current standard of care diagnostics to maximize both sensitivity and specificity.

### Clinical outcomes and utility

The goal of earlier detection is improved patient outcomes. While direct evidence of mortality reduction via ctDNA-based screening is not yet available, early diagnosis consistently correlates with better prognosis in analogous cancers such as colorectal cancer (61). In EC, a shift from stage III to stage II at diagnosis could allow for curative surgery in an otherwise unresectable case.

ctDNA may also detect cancers prior to clinical symptoms. In a small study by Nasrollahzadeh et al., TP53 mutations were found in 5 of 39 asymptomatic individuals, of which one was diagnosed with ESCC 6 months later while the others remained cancer-free during follow-up (47). This suggests ctDNA may capture early neoplastic signals although distinguishing true positives from background mutations like CHIP remains a challenge.

Beyond initial diagnosis, ctDNA has demonstrated clinical utility in post-treatment surveillance. Patients who have undergone curative esophagectomy or definitive chemoradiotherapy can be monitored for continued undetectable ctDNA. The reappearance of ctDNA typically precedes radiologic or symptomatic relapse by several months, with one study reporting a median lead time of 4.1 months over routine imaging (27, 62–66). Such early detection of recurrence could allow prompt intervention at a minimal disease state, potentially improving outcomes. While surveillance differs from initial screening, it reinforces the clinical utility of ctDNA as an early warning tool in EC management.

In summary, ctDNA as a liquid biopsy represents a promising tool for earlier detection of EC, with growing evidence supporting its diagnostic accuracy and prognostic significance. However, sensitivity limitations in stage 0-I remain a key barrier to widespread clinical adoption. Survival impact is not fully demonstrated as ctDNA screening is not standard of care. Ongoing research is focused on improving assay sensitivity, validating biomarkers prospectively, and defining ctDNA’s role within multi-modal screening practices.

## Stratification of ctDNA utility in esophageal adenocarcinoma and esophageal squamous cell carcinoma

EAC and ESCC differ markedly in their epidemiology, etiology, and molecular landscape (**Table 1**). These differences also extend to ctDNA characteristics through shedding behavior and mutational profiles. Stratifying ctDNA applications by histologic subtype will be important as emerging evidence suggests that optimal detection may vary between EAC and ESCC.

**Table 1:** Features of EAC and ESCC.

Features	EAC	ESCC
Common Risk Factors	Barrett’s esophagus, GERD	Tobacco use, alcohol consumption, dietary nitrosamines
Epidemiology	North America and Europe	Asia, South America, Africa
Pathology	Squamous epithelium replaced by columnar epithelium	Squamous dysplasia
Location	Distal esophagus	Proximal to middle esophagus
Genomic Alterations	*TP53, CDKN2A, KRAS, HER2* amplifications	*TP53, KMT2D, NOTCH1, NFE2L2, PIK3CA, PTEN, RB1, SOX2, PD-L1* amplifications
ctDNA Shedding	Generally higher at early stages	Often lower at very early stages
Sensitivity of ctDNA for Early Detection	36%–47%	33%–50%

ctDNA, circulating tumor DNA; EAC, Esophageal adenocarcinoma; ESCC, Esophageal squamous cell carcinoma; GERD, gastroesophageal reflux disease.

### Tumor biology, genomic profiles, and ctDNA shedding

EAC is commonly seen in patients with Barrett’s esophagus and chronic gastroesophageal reflux disease (GERD) whereas ESCC is strongly associated with tobacco use, alcohol consumption, and dietary exposures. EAC is more commonly observed in North America and Europe while ESCC is more commonly observed in Asia, South America, and Africa. Histologically, EAC tends to invade the submucosa, while ESCC typically remains confined to the mucosa.

Genomically, EAC more commonly harbors mutations in *KRAS, TP53*, and *CDKN2A* and demonstrates higher *HER2* expression. In comparison, ESCC shows a more distinct mutation profile in *KMT2D, NFE2L2, NOTCH1, PIK3CA, PTEN, RB1, SOX2*, and *TP53*, and demonstrates higher *PD-L1* expression (67, 68).

These biological differences may influence ctDNA shedding as EAC tumors are believed to shed ctDNA more abundantly than ESCC tumors. This may be due to differences in vascular invasion rates and tumor cell turnover​. As a result, ctDNA-based detection may be more sensitive for EAC. However, several studies have shown modest differences in sensitivity between EAC and ESCC, suggesting that additional factors such as assay design and disease stage may also contribute to ctDNA detection sensitivity.

### Clinical outcomes and utility

Targeted ctDNA panels have potential clinical utility when applied to individuals at high risk for EAC or ESCC. However, targeted ctDNA studies often focus on predefined mutations and may miss key driver mutations, leading to false negatives.

When stratified by histology, ctDNA sensitivity for early-stage detection does not demonstrate significant differences between EAC and ESCC​​ (49, 62, 69–71). Regardless, the distinct molecular profiles of these EC subtypes suggest that histology-specific or multi-marker models may enhance performance and detection. Integrating histology into machine learning models interpreting ctDNA signals could further improve early detection.

## Analysis of prior systematic reviews and meta-analyses

Several systematic reviews and meta-analyses have synthesized the growing evidence on ctDNA in EC, offering pooled estimates of diagnostic performance, prognostic value, and assay variability. **Table 2** summarizes key findings, stratified where possible by histology and clinical context.

**Table 2:** Summary of systematic reviews and meta-analyses of EC stratified into EAC and ESCC.

Study (Year)	Included studies (Patients)	Context	Sensitivity (%)	Specificity (%)	HR	OS/CI/p-value	OS (HR) - EAC	OS (HR) - ESCC	Key Findings
Chidambaram et al. (2022) (21)	15 studies (414 patients); 8 in meta-analysis	Diagnosis & surveillance of EC	71.0% (95% CI 55.7%–82.6%) (Diagnosis) 48.9% (95% CI 29.4%-69.8%) (Surveillance)	98.6% (95% CI 33.9%–99.9%) (Diagnosis) 95.5% (95% CI 90.6%–97.9%) (Surveillance)	Not assessed	Not reported	Not reported separately	Not reported separately	ctDNA shows moderate sensitivity and high specificity in EC diagnosis; sensitivity lower in post-treatment surveillance. Detection improved when combined with imaging.
Zhang et al. (16) (72)	13 studies (604 patients)	Prognostic value of ctDNA in EAC and ESCC	Not reported for diagnosis	Not reported for diagnosis	HR for OS: 3.65 (95% CI 1.97–6.75) HR for DFS/RFS: 6.08 HR for PFS: 2.84	OS significantly worse in ctDNA-positive patients p < 0.001 across endpoints	HR: 6.74 (95% CI: 3.50–12.98)	HR: 2.84 (95% CI: 1.26–6.42)	ctDNA positivity predicts worse outcomes in EC, across histologic types and treatment contexts. Stronger prognostic effect in EAC than ESCC.
Bittla et al. (2023) (48)	14 studies (multiple cancer types)	Early cancer detection (multi-cancer context)	69%–98% (range across tumor types)	~99%	Not assessed	Not reported	Not applicable	Not applicable	ctDNA highly specific but sensitivity depends on tumor type and size; limited utility for tumors <1 cm. More useful for monitoring and prognosis than for screening.
Xu et al. (2024) (73)	22 studies (1,144 patients)	Prognostic value of ctDNA in EC	Not reported for diagnosis	Not reported for diagnosis	3.87 (95% CI 2.86–5.23)	P < 0.001	Not reported separately	Not reported separately	ctDNA positivity associated with decreased OS and PFS in EC patients. Both ddPCR and NGS platforms effective for ctDNA detection. Highlights need for standardization in ctDNA analysis for clinical application.

CI, Confidence interval; ctDNA, circulating tumor DNA; ddPCR, digital droplet PCR; DFS, Disease-free survival; EC, Esophageal cancer; EAC, Esophageal adenocarcinoma; ESCC, Esophageal squamous cell carcinoma; HR, Hazard ratio; NGS, Next-generation sequencing; OS, Overall survival; PFS, Progression-free survival; RFS, Recurrence-free survival.

In 2022, Chidambaram et al. conducted one of the first comprehensive meta-analyses examining ctDNA in EC, including 15 studies (414 patients), of which 8 studies were utilized in a pooled analysis​ (21). The authors reported a pooled diagnostic sensitivity of 71.0% and specificity of 98.6% for ctDNA-based detection of EC, combining various ctDNA assay types. Sensitivity was markedly higher for detection of established cancers than for post-treatment surveillance of recurrence. In contrast, ctDNA used for post-treatment surveillance demonstrated a pooled sensitivity of 48.9% and specificity of 95.5%, likely reflecting small-volume or local recurrences. ctDNA performance improved when combined with imaging, supporting its complementary role rather than standalone use, emphasizing the need for assay standardization and clinical integration.

In 2024, Zhang et al. analyzed 13 studies (604 patients) to evaluate the prognostic value of ctDNA in EC (72). The results demonstrated ctDNA positivity was associated with worse outcomes. The pooled hazard ratio (HR) for OS for ctDNA-positive patients vs. negative was 3.65 (95% CI 1.97–6.75), while HRs for disease-free survival/recurrence-free survival and progression-free survival were 6.08 and 2.84, respectively. Subgroup analyses by EAC and ESCC, timing of ctDNA detection pre- or post-treatment, and assay method, confirmed these trends. These findings suggest that detectable ctDNA reflects higher tumor burden or early relapse, highlighting the prognostic weight a positive result may carry. From an early detection standpoint, these data highlight the importance of maintaining high specificity to avoid unnecessary intervention or anxiety.

### Other reviews

A 2023 systematic review by Bittla et al. evaluated ctDNA for early cancer detection across multiple tumor types, providing context relevant to EC despite not being tumor-specific​ (48). The authors reported that ctDNA assays have limited utility for tumors less than 1 cm in size and emphasized that an effective early screening test would need to detect tumors less than 5 mm in size, which is below current detection thresholds. Reported values for ctDNA-based detection across cancers ranged from 69%–98% sensitivity and 99% specificity depending on tumor type and assay​. The authors concluded that ctDNA is currently more reliable as a prognosis and disease monitoring tool than for primary early detection given its strong correlation with outcomes and the challenges in capturing the smallest tumors​. These findings reinforce the need for technologic improvements before ctDNA can be widely utilized to screen asymptomatic individuals.

A 2024 narrative review by Xu et al. examined cfDNA methylation assays, highlighting its promise as a non-invasive method for early EC ​detection and its need for improved sensitivity and validation (73). The authors noted that cfDNA methylation assays mostly focused on Barrett’s esophagus associated EAC with fewer data on ESCC. The review concluded that cfDNA methylation detection holds significant potential as an early EC detection test, aligning with current ctDNA assay development.

Collectively, these systematic reviews support ctDNA as a credible biomarker for both diagnosis and prognosis in EC. However, they also consistently emphasize its limited sensitivity for detecting the smallest or earliest-stage tumors. All reviews call for larger prospective studies to validate the use of ctDNA in the setting of EC screening or early diagnosis as existing evidence is largely retrospective or observational in already-diagnosed patients. The following section outlines the technical and clinical challenges that need to be addressed to move ctDNA from a promising research tool into a reliable screening tool.

## Technical and clinical challenges

Implementing ctDNA-based early detection for EC faces several technical and clinical challenges. These challenges must be acknowledged and overcome to ensure ctDNA testing is accurate, cost-effective, and clinically meaningful.

### Sensitivity limitations and low tumor shedding

The foremost technical challenge is the limited sensitivity of ctDNA for detecting very small tumors or early-stage tumors. Tumors under 1–2 cm in size or intra-mucosal lesions may shed minimal ctDNA, falling below detection thresholds and evading detection​ (21, 48). Even ultra-deep sequencing may fail to detect tumors if only a limited number of tumor cells are undergoing apoptosis and releasing DNA.

Improving sensitivity will likely require multi-modal approaches, such as combining genomic mutations with epigenomic and fragmentomic features, enriching tumor-specific fragments by size or methylation, and applying advanced error-correction techniques. For example, in stage 0/I ESCC, adding low-pass whole-genome sequencing fragmentation features to 5hmC profiling increased accuracy from 33% to 80% for carcinoma *in situ*, demonstrating the promise of combined modalities​ (45).

### False positives and specificity issues

While ctDNA assays generally have high analytical specificity, biological false positives remain a major concern, particularly from CHIP. As individuals age, more somatic mutations in genes such as *TP53, DNMT3A*, and *TET2* can shed mutated DNA into plasma, mimicking tumor-derived mutations. In one study, 11% of individuals without cancer had detectable cancer-like *TP53* mutations in plasma, even after filtering for known CHIP-associated mutations, and most individuals did not develop any malignancy (46).

Distinguishing true tumor-derived ctDNA from CHIP-related background noise is crucial to avoid unnecessary procedures or anxiety. Approaches include sequencing matched white blood cells to identify CHIP mutations and prioritizing methylation or fragmentomic patterns that are more tumor-specific. Maintaining near-100% specificity is essential for screening, even if it requires sacrificing some sensitivity.

### Tumor heterogeneity and assay design

ECs exhibit significant heterogeneity with different regions harboring distinct mutations. A ctDNA assay that only targets a few specific mutations may not detect a tumor lacking those mutations. For example, a panel designed around common EAC mutations like *TP53* or *CDKN2A* could miss detection of an ESCC that primarily has *NOTCH1* or *PIK3CA* mutations, and vice versa (34, 74).

Broader NGS panels or non-targeted methods can capture a wide array of possible tumor signals, but at the expense of higher cost and complexity (75, 76). Another approach is a tumor-informed strategy for high-risk patients, sequencing mutations identified from the patient’s lesion or cancer​ (77). This has been shown to greatly enhance detection of residual disease or recurrence in EC (17, 27, 62–65), but is only applicable when a tumor sample is available.

### Standardization of techniques

A major challenge is the lack of standardization across ctDNA platforms. As highlighted in the Chidambaram meta-analysis, no studies have directly compared ddPCR and NGS head-to-head in EC (21). NGS offers broad mutational coverage and discovery power, while ddPCR may achieve superior sensitivity for known mutations (42, 78). However, ddPCR requires individualized assay design, limiting scalability.

A potential solution is a two-step model utilizing NGS for initial profiling followed by patient-specific ddPCR for surveillance. Regardless of platform, standardization in sample collection, processing, and interpretation is essential. Efforts like the Plasma-Seq consortium (79) aim to establish shared protocols. Without standardization, variability will hinder regulatory approval, clinical adoption, and payer reimbursement, especially given the high cost of advanced sequencing.

### Cost-effectiveness and public health feasibility

Cost remains a significant barrier to large-scale implementation of ctDNA screening. High-sensitivity assays, particularly those using ultra-deep sequencing or methylation profiling, often cost several hundred dollars per test. Healthcare systems must weigh these costs against demonstratable benefits in earlier EC detection and improved outcomes. However, in the Endoscopic Screening for Esophageal Cancer in China (ESECC) trial, a precision screening strategy based on individualized risk stratification and targeted endoscopy reduced the cost per detected case from approximately $14,944 to $7,148–$11,537 in a high-risk Chinese population, suggesting that a personalized approach may have feasible economic benefits (80).

In the US, a recent cost-effectiveness analysis evaluated a multi-cancer early detection (MCED) strategy incorporating cfDNA methylation. The results showed an incremental cost-effectiveness ratio (ICER) of $66,048 per quality-adjusted life year (QALY), well within commonly accepted thresholds of less than $100,000 per QALY. Although upfront testing costs were substantial, long-term savings were projected due to a shift toward earlier-stage diagnoses, reduced treatment intensity, and improved survival (81). While this analysis utilized cfDNA methylation, the principle remains that multi-modal approaches to EC screening may have feasible economic benefits rather than ctDNA testing alone.

The cost of diagnostic upper endoscopy for EC ranges from $1,000-$3,500 in the US depending on setting and insurance coverage. Although endoscopy remains the gold standard for diagnosis, it is invasive, resource-intensive, and often impractical for large-scale screening of asymptomatic individuals. Additional limitations include the need for scheduling, anesthesia, and procedural risks. ctDNA offers a non-invasive alternative but must demonstrate comparable or superior cost-effectiveness in high-risk populations to justify widespread implementation.

The broader oncology literature supports the economic value of early detection, as late-stage cancer care is often nearly twice as expensive as early-stage treatment, with added burdens of prolonged hospitalization and more aggressive therapy.

Infrastructure and system readiness also remain obstacles. Widespread ctDNA screening would require standardized protocols for blood collection, processing, sequencing, and interpretation, elements that are not universally established yet. Equitable deployment will additionally require addressing disparities in diagnostic follow-up and access to endoscopy and subspecialty care, particularly in underserved or resource-limited settings.

In summary, while early modeling supports the cost-effectiveness of ctDNA-based screening in high-risk groups, public health implementation will require further prospective validation, infrastructure investment, and payer reimbursement strategies to ensure sustainable adoption.

### Clinical trial data and endpoints

Demonstrating clinical benefit is essential for adding ctDNA screening or surveillance to standard of care. Evidence is needed to show that ctDNA either detects cancers that would have been missed by standard screening or detects them earlier, resulting in improved outcomes. However, designing such trials is challenging.

For screening, a potential approach would be randomizing high-risk individuals to receive ctDNA tests vs. no ctDNA tests and undergoing endoscopic screening at pre-specified intervals for both groups. Endpoints would include reduced incidence of advanced EC in the ctDNA arm group. However, sample sizes would have to be large given the relatively low incidence of EC in the Western population. Focusing on high-risk groups such as patients with Barrett’s esophagus, tobacco users/alcohol consumers, and patients from high-incidence regions like East Asia may make studies more feasible (82, 83).

Another clinical question is how to manage a positive ctDNA result when endoscopy and imaging are negative as the positive ctDNA could reflect early disease, or a false positive. Surveillance, repeat testing, or empirical intervention may be considered, but one would need to consider patient anxiety, risk, and costs involved. Furthermore, some early EC may be indolent or detected later by standard of care screening, raising concerns about lead-time bias. Thus, demonstrating meaningful clinical endpoints is a challenge that will require well-designed prospective studies.

### Integration with existing pathways

Clinicians will need clear guidelines on how to interpret and act on ctDNA results. For example, if a patient with known Barrett’s esophagus develops a TP53-positive ctDNA signal, should this prompt immediate endoscopic mucosal resection? In a post-esophagectomy surveillance patient, how should ctDNA results influence follow-up scheduling or initiation of therapy?

Early studies have shown that clinicians can use rising ctDNA levels to predict recurrence and guide earlier intervention, but these strategies remain investigational. Regulatory approvals and insurance coverage will require demonstrated clinical validity and utility as payers may not cover expensive ctDNA assays for early detection purposes. Until then, ctDNA may serve best as a complementary tool rather than a replacement for current standard screening.

In summary, while ctDNA holds significant promise, several technical hurdles and clinical challenges must be overcome. Addressing low ctDNA levels in early-stage tumors, false positives from CHIP, tumor heterogeneity, and the need for standardized, cost-effective assays are important. Multi-modal strategies and advanced sequencing methods may improve sensitivity while improved analysis can help distinguish true tumor signals from background noise.

To translate ctDNA into a reliable screening tool, prospective studies must demonstrate improved outcomes, not just earlier detection. Public health implementation will also require infrastructure investment, payer coverage, and clear clinical guidelines. Continued research and validation will be essential to realize ctDNA’s full potential in EC care.

## Future perspectives and clinical integration

Looking forward, the landscape of ctDNA in EC is likely to evolve in three key areas: technological enhancements, large-scale validation studies, and integration into multidisciplinary cancer care. Below, we outline future directions and how ctDNA might be incorporated into routine clinical practice.

### Technological innovations

Continued advances in sequencing technologies and bioinformatics are expected to improve the sensitivity of ctDNA assays. Ultrasensitive error-corrected sequencing platforms, including CAPP-Seq, Safe-SeqS, and tagged-amplicon deep sequencing, can reduce error noise and detect mutant allele fractions below 0.01%, offering promise for early-stage EC (84–86). In parallel, machine learning is being explored to interpret cfDNA fragment characteristics, such as fragment size distributions and end motifs, as another way to distinguish cancer-derived DNA from normal cfDNA. Emerging research continues to support combining multiple features, such as mutations, methylation patterns, and fragmentation profiles, to ultimately improve detection sensitivity and specificity. For example, integrating methylation markers with fragmentation signatures and mutation data has achieved high diagnostic accuracy in research settings​​ (45).

In the future, a single blood test could perform targeted sequencing for a panel of mutations, assess methylation, and profile ctDNA to generate a unified EC risk score. In addition, improvements in bioinformatics will help filter out CHIP-related mutations and other false signals more effectively, possibly by referencing large databases of common CHIP mutations and typical error patterns. To make widespread use feasible, technological advances must also focus on cost reduction and workflow automation, key factors for population-level screening beyond oncology specialty centers.

### Multi-Cancer Early Detection and Screening Programs

There is growing interest in ctDNA-based MCED tests to identify EC as no established screening program exists outside of high-risk populations. Tests like the Galleri assay, which uses cfDNA methylation profiles, are being evaluated in prospective studies although specific performance for EC has not been published. A potential future scenario could involve ctDNA testing during routine check-ups for patients with high risk factors such as chronic GERD or a history of tobacco and alcohol use. A positive ctDNA result would trigger further evaluation with endoscopy or imaging. Ongoing trials such as the PATHFINDER study (87) are exploring the clinical implementation of such testing; results will inform the feasibility, false positive rates, and stage distribution of detected cancers.

### Integration into clinical workflows

If validated, ctDNA testing could enhance several stages of EC care. For high-risk patients with longstanding Barrett’s esophagus or head and neck cancer survivors at risk for ESCC, periodic ctDNA screening could complement standard surveillance. In newly-diagnosed EC patients, a baseline ctDNA analysis could be performed for both risk stratification and identification of actionable mutations to track. During treatment, serial ctDNA measurements may reflect tumor response and help guide therapy adjustments (88).

For post-treatment, ctDNA monitoring every 3–6 months could be integrated into follow-up guidelines to detect minimal residual disease or early recurrence before radiographic or symptomatic progression. As clinical adoption of ctDNA testing expands, oncologists and gastroenterologists may become more confident interpreting these results. Multidisciplinary tumor boards may routinely review ctDNA results when making decisions about surveillance plans or additional treatment.

### Regulatory and economic consideration

To integrate ctDNA into clinical practice, tests will need regulatory approval and guideline endorsement, requiring demonstration of analytical and clinical validity. Economic evaluations will need to show that ctDNA testing is cost-effective, especially compared to endoscopy, in high-risk groups.

As technology improves, costs are expected to decrease, making widespread ctDNA screening more economically feasible. However, questions around incidental findings, such as detecting colorectal cancer in an EC screening, informed consent, and follow-up management will require ethical and logistical frameworks. Additionally, clear reimbursement policies and equitable access must be addressed to ensure responsible implementation.

The future of ctDNA in early EC detection is promising. For a disease with no standardized screening protocol except in limited high-risk groups, ctDNA-based approaches could be transformative, enabling personalized, precision oncology through detecting EC based on its unique molecular fingerprints even before it causes a structural lesion visible on endoscopy. To reach that goal, ongoing research is focusing on maximizing detection of that first sign of tumor DNA in the blood.

## Divergent pathways in early esophageal cancer detection: the role of ctDNA and esophagogastroduodenoscopy in screening strategies

Esophagogastroduodenoscopy (EGD) remains the gold standard for EC detection, offering direct visualization, biopsy, and histological confirmation. However, it is invasive, expensive, and impractical for mass screening. ctDNA, by contrast, offers a non-invasive blood-based approach that can detect molecular traces of cancer before clinical symptoms or visible lesions appear. It holds promise for broader, earlier screening but remains a challenge regarding sensitivity in very early-stage disease, standardization, and cost-effectiveness. Emerging studies suggest ctDNA might eventually complement or even reduce reliance on EGD for screening high-risk populations. However, ctDNA is not currently ready to replace EGD as a standalone diagnostic tool.

## Conclusions

EC remains a formidable clinical challenge due to late-stage diagnosis and poor prognosis, but the advent of ctDNA analysis offers hope for changing that paradigm. ctDNA is a viable biomarker for early detection of EC, demonstrating high specificity for tumor presence and the capacity to non-invasively detect a significant subset of early-stage tumors. Studies consistently show that ctDNA positivity reflects tumor burden and is associated with worse outcomes, highlighting its clinical significance. Modern ctDNA assays, especially those leveraging tumor-specific methylation patterns or patient-tailored mutation panels, have pushed sensitivity to new heights, detecting about 70-75% of stage I–II EC in initial studies​ (53). This level of performance, while not yet perfect, suggests that ctDNA could become a useful adjunct to endoscopic screening and a tool for identifying asymptomatic cancers at a curable stage.

Translating ctDNA early detection into routine clinical practice requires surmounting key challenges. Technical improvements are needed to ensure that microscopic tumors can be reliably detected, minimizing false negatives without generating false positives from background mutations. The current literature underscores a trade-off between sensitivity and specificity that future assays must balance. Moreover, standardized guidelines are needed to determine how a positive ctDNA test should trigger confirmatory diagnostics and influence management. Interdisciplinary collaboration between oncologists, gastroenterologists, surgeons, and pathologists will be essential to integrate ctDNA testing protocols in a manner that complements existing standards of care.

In conclusion, ctDNA-based early detection for EC is approaching clinical reality, driven by rapid innovation and expanding evidence. For a disease that has long lacked a convenient screening method, ctDNA offers a minimally invasive approach that could enable earlier diagnosis, personalized treatment decisions, and improved outcomes. Ongoing large prospective trials and real-world implementation studies are the next steps to demonstrate that ctDNA testing could revolutionize the proactive management of EC. Clinicians could detect EC at its molecular inception and intervene when cure is achievable, shifting the narrative of EC from late-stage discovery to early-stage interception, ultimately changing the poor outcomes and statistics that have plagued this disease for decades.
